# Sleep Reactivity and Depressive Symptoms Among Chinese Female Student Nurses: A Longitudinal Mediation Analysis

**DOI:** 10.3389/fpsyt.2021.748064

**Published:** 2021-09-29

**Authors:** Xuliang Shi, Haiying Qi, Shuo Wang, Zihan Li, Zhipeng Li, Fang Fan

**Affiliations:** ^1^Hebei University, Baoding, China; ^2^South China Normal University, Guangzhou, China

**Keywords:** sleep reactivity, depressive symptoms, sleep disturbance, emotion regulation difficulties, shiftwork

## Abstract

Previous cross-sectional studies have documented that sleep reactivity was associated with depressive symptoms, but the potential mechanisms underlying this relationship were understudied. Therefore, the present study with a longitudinal prospective design was to reveal the mediating roles of sleep disturbance and emotion regulation difficulties (ERD) between sleep reactivity and depressive symptoms. This study included 725 student nurses who were followed up periodically for 9 months, with an interval of three months. All participants completed questionnaires regarding sleep reactivity, sleep disturbance, ERD, and depressive symptoms. Adjusted analyses suggested that the direct effect of sleep reactivity on depressive symptoms was non-significant. The bootstrap procedure revealed two significant indirect effects: from sleep reactivity to depressive symptoms with sleep disturbance as a mediator and from sleep reactivity to depressive symptoms with sleep disturbance and ERD as sequential mediators. Therefore, sleep reactivity might be considered as an indicator of shiftwork adaptability in the evaluation of recruitment. Psychological interventions aimed at developing healthy sleep habits and emotion regulation skills may be helpful in decreasing the risk of depression.

## Introduction

Almost all of the student nurses have to work in shifts when they enter in the internship position. Shiftwork can be regarded as a stressor that disrupts their normal sleep-wake patterns and causes circadian rhythm misalignment (1). Some student nurses can adapt to shift work within a period of time, while others cannot adjust well and thus lead to negative health conditions, such as sleep disturbance, depression, and anxiety (2). A recent cross-sectional study in China (*n* = 1592) found that 25.1% of nurses reported moderate to severe depressive symptoms (3). Similar results have been reported among nurses in South Korea (4). Without timely intervention and treatment, transient depressive symptoms may exert long-term adverse effects on individual mental health including suicidal thoughts and behaviors (5). Therefore, at-risk student nurses must be identified prior to the development of depression.

Sleep reactivity is defined as the extent to which sleep is disrupted in response to stress exposure (6, 7), reflecting an individual's vulnerability to experience situational insomnia. It is commonly considered to be a predisposing factor to insomnia (6). Individuals with high sleep reactivity are prone to transient sleep disturbance and increased wake-time sleepiness in response to a single night of circadian misalignment (7). Sleep reactivity may not only be a trait vulnerability to shiftwork-related sleep disturbance but also to shiftwork-related changes in depression. However, inconsistent results were found regarding whether sleep reactivity was directly related to the development of depression or if this relationship can be mediated by sleep disturbance. For example, Vargas et al. used data from Colorado Longitudinal Twin Study and Community Twin Study (*n* = 2250), and found that sleep reactivity was independently associated with depressive symptoms, and this link was partially mediated by insomnia (8). However, in another study of pregnant women (*n* = 62), researchers found that sleep reactivity was not directly associated with depressive symptoms when accounting for the effects of anxiety and insomnia (9). These results need to be further confirmed with more robust methodologies such as a longitudinal prospective design with large sample sizes.

According to Gratz and Roemer (10), emotion regulation is referred to the “ability to act effectively in the context of emotionally salient events”, which includes four key aspects: (a) awareness and identification of emotions; (b) acceptance of emotions; (c) controlling for impulse and engaging in goal-directed behaviors when experiencing negative emotions; and (d) the ability to use emotion regulation strategies flexibly. Recent studies have demonstrated that emotion regulation abilities were important predictors of affect, cognition, and behaviors (11). The association between emotion regulation difficulties (ERD) and depression has been well-established. Individuals who are prone to depression may have difficulty in understanding emotions and have limited ability to tolerate them. Clinical studies have confirmed that compared with healthy controls, depressed individuals reported a more frequent use of negative emotion regulation strategies, such as rumination, suppression, and catastrophizing, and a less frequent use of positive strategies, such as cognitive reappraisal and self-disclosure (12, 13). Besides, emotion regulation abilities may be affected by sleep loss or sleep disruption in the aspects of understanding, expressing, and modifying negative emotions (14, 15). In a previous lab-based study, researchers found that even several nights of shortened sleep can result in worsened mood and emotion regulation in adolescents (16). Congruently, a study with functional magnetic resonance imaging (fMRI) showed that one night of sleep deprivation alone triggered an obvious reduction in functional connectivity between the medial prefrontal cortex and the amygdala, which was considered as an important component of emotion regulation (17). In addition, the cross-sectional (18, 19) and longitudinal studies (20) provided preliminary evidence that sleep disturbance impaired the ability to access and leverage emotion regulation strategies, which in turn increased the risk of depression.

The present study extends previous research in two important aspects. First, prior studies were mostly cross-sectional in design, making it difficult to determine the causal relationship between sleep reactivity and depressive symptoms. Second, no prior study has tested an integrative model that involves both sleep disturbance and ERD in examining the roles of sleep reactivity in the development of depression.

## Materials and Methods

### Participants and Procedures

**Figure 1:**
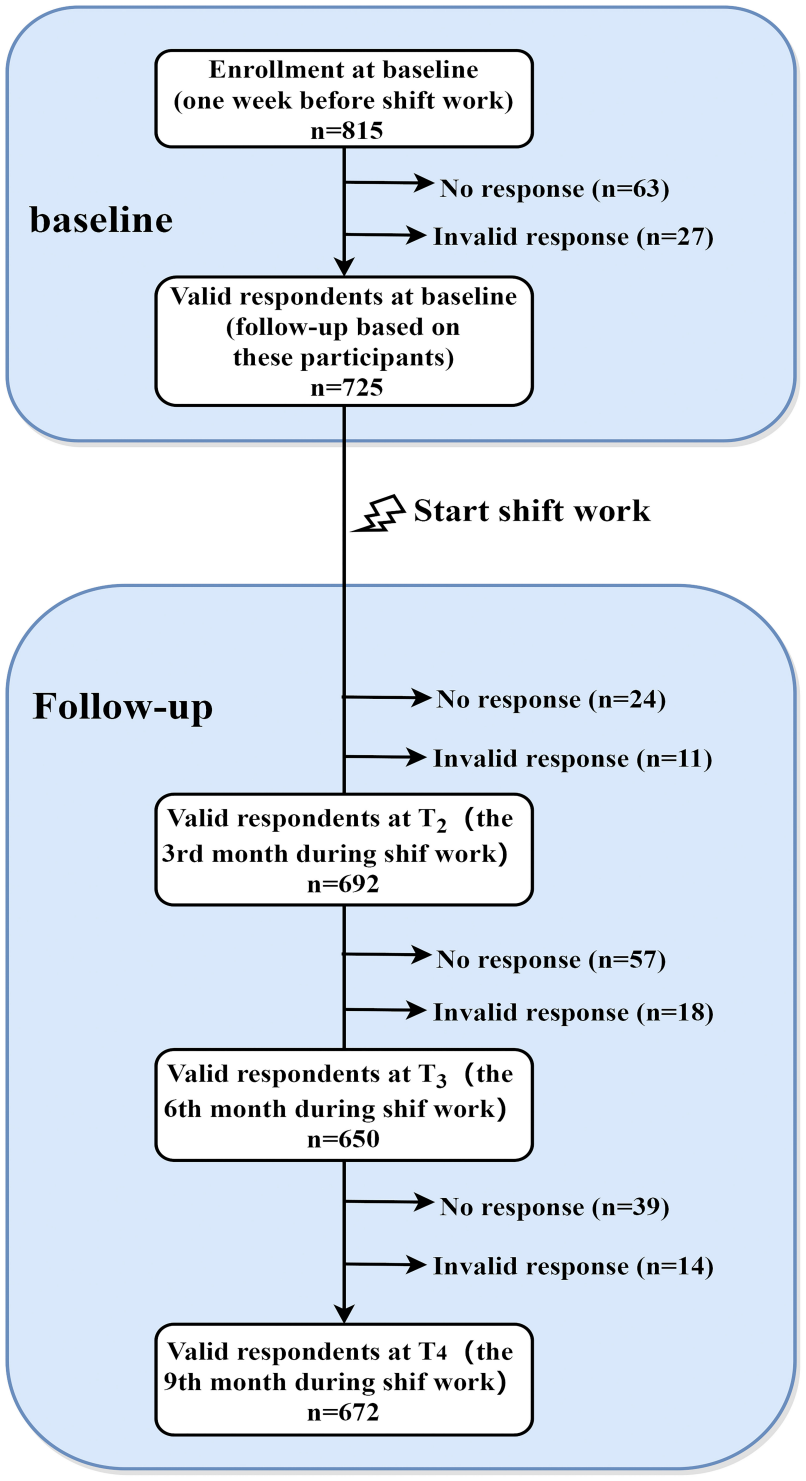
Sampling procedure for the current study.

All participants without any shift experience were recruited from a nursing school in Guangzhou, China. At baseline (one week before they transited to internship in hospital, T_1_), a total of 815 third-grade students were invited to participate in the online survey, and 752 participants completed the survey (the response rate = 92.27%). After removing the invalid data (*n* = 27), 725 eligible participants (mean of age at baseline = 17.8 years, SD = 1.52) were resurveyed in the follow-up investigations. Participants were excluded if their response time was less than 10 minutes or their responses included invalid answers. In brief, participants were reassessed at the 3^rd^ month (T_2_), the 6^th^ month (T_3_), and the 9^th^ month (T_4_) during the shift work in hospital. At T_2_, T_3_ and T_4_, a total of 692 (95.45%), 650 (89.66%) and 672 (92.69%) of the T_1_ participants retained, respectively. Detailed sampling procedure has been depicted in Figure 1.

Data were collected through an online website that was designed and maintained by our research team. All participants completed their first survey at school, and the follow-up surveys were completed in hospital. Before the assessment, school authorities and the head teachers were informed of the purpose and contents of this study. After that, the head teachers sent the questionnaire to student nurses through a mobile social networking app (i.e. WeChat). Participants were firstly asked to read the instructions carefully and their participation was voluntary. If they agreed to participate, they could choose “yes” and then continued finishing the following parts. Otherwise, the investigation would stop immediately. After the survey, the head teachers would count the number of students who have completed the survey. Two methods were adopted to make sure the participants completed the survey carefully. First, a unified training for the head teachers were conducted before data collecting to ensure the consistency of guidelines. Second, two screening criteria were used to test whether the participants answered carefully, including the total answer time auto-recorded in the online system and two questions that to detect discrepancies. During the survey, all participants were informed that they can withdraw at any time if they feel uncomfortable.

Ethical approval for this study was obtained from the Human Research Ethics Committee of South China Normal University, with permission and support from the participating school boards. An electronic version of the informed consent form was obtained from all participants.

### Measures

At baseline (T_1_), socio-demographical variables, sleep reactivity, sleep disturbance, emotion regulation difficulties, and depressive symptoms were measured. At T_2_, shiftwork-related variables and sleep disturbance were measured. At T_3_, and T_4_, we repeatedly evaluated emotion regulation difficulties and depressive symptoms, respectively.

### Sleep Reactivity

The Chinese version of FIRST (Ford insomnia response to stress test, FIRST) was used to assess the likelihood of sleep disturbance when exposed to stressful situations (21). Samples of questions as follows: “When you experience the following situations, how likely you will have difficulty in sleeping?”, such as (1) “Before an important meeting the next day” and (2) “After an argument”. This scale includes nine items with response options anchored on a 4-point Likert scale ranging from 1 (*not very likely*) to 7 (*very likely*). Scores for all items were summed to yield a total score ranging from 9 to 36. A higher total score indicates a higher level of sleep reactivity to the situations. This scale has been widely used in Chinese population and demonstrated adequate reliability and validity (21, 22). In the present study, Cronbach's alpha was 0.87 at T_1_.

### Sleep Disturbance

Four items adapted from the Pittsburgh Sleep Quality Index were used to assess self-reported sleep disturbance during the past three months: (1) How would you rate your sleep quality overall? (2) How often have you had trouble sleeping because you cannot get to sleep within 30 min at night (difficulty initiating sleep, DIS)? (3) How often have you had trouble sleeping because you wake up frequently during the night (difficulty maintaining sleep, DMS)? and (4) How often have you had trouble sleeping because you wake up very early (early morning awakening, EMA)? The first item included 4 choices: 0 = excellent; 1 = good; 2 = poor; and 3 = very poor. The last three items were scored as follows: 0 = never; 1 = less one night per week; 2 = one or two nights per week; and 3 = three nights or more per week. For item 1, a response of “poor” or “very poor” was considered as having poor sleep quality. For items 2, 3, and 4, a frequency of at least three times per week indicated DIS, DMS, and EMA, respectively. Four items were added to create a total score ranging from 0 to 12, with higher scores indicating higher levels of sleep disturbance. These items have been used extensively in previous studies (23–25). In the present sample, Cronbach's alpha was 0.79 at T_1_ and 0.82 at T_2_.

### Emotion Regulation Difficulties

Emotion regulation difficulties (ERD) were assessed by a brief version of the Difficulties in Emotion Regulation Scale (DERS-16) (26). It comprises 16 items answered on a five-point Likert scale ranging from 1 (*almost never*) to 5 (*almost always*). The scale was classified into five dimensions, including: non-acceptance of emotional responses, difficulties engaging in goal-directed behaviors, impulse control difficulties, limited access to effective emotion regulation strategies, and a lack of emotional clarity. Scores from 16 items were summed to get a total score ranging from 16 to 80, with higher scores indicating a higher level of emotion dysregulation. The Chinese version of DERS-16 was translated by our research team. In the current study, confirmatory factor analysis was conducted to examine the construct validity of the DERS-16, and the results showed that the data fitted well (χ^2^ = 468.04, *df* = 94, *p* < 0.001, CFI = 0.94, TLI = 0.91, RMSEA = 0.08). In the present sample, Cronbach's alpha was 0.94 at T_1_ and 0.95 at T_3_.

### Depressive Symptoms

Depressive symptoms in the past two weeks were evaluated by the Chinese version of Patient Health Questionnaire (PHQ-9) (27). The PHQ-9 comprises nine diagnostic criteria of the Diagnostic and Statistical Manual of Mental Disorders, 4^th^ Edition (DSM-IV) for depression. It was a four-point Likert scale from 0 (*not at all*) to 3 (*nearly every day*) and a total score from 0 to 27 was calculated. The PHQ-9 score (range: 0–27) was divided into the following categories of increasing severity: 0–4 (minimal depression), 5–9 (mild depression), 10–14 (moderate depression), 15–19 (moderately severe depression) and 20–27 (severe depression). Probable depression was indicated by a cut-off score of 10. The PHQ-9 has also been demonstrated to have good psychometric properties in the general population (27, 28). In the present sample, Cronbach's alpha was 0.86 at T_1_ and 0.94 at T_4_.

### Covariates

Socio-demographical variables at T_1_ (BMI, siblings, residence, exercise habit, current alcohol use, parents' marital quality, and family income) and shiftwork-related variables at T_2_ (shift schedule, night shift nap, and pressure during shiftwork) were used as covariates.

### Statistical Analyses

Little's Missing Completely at Random (MCAR) test was used to clarify the missing data mechanism, and the results indicated that the data were missing at random (MAR), χ^2^ = 204.01, *df* = 181, *p* = 0.12. In addition, Chi-square and *t* test were also used to compare the differences in socio-demographical and shiftwork-related variables for participants who completed all surveys with those who did not, and the results showed no differences in BMI, siblings, residence, exercise habit, current alcohol use, parents' marital quality, family income, and night shift nap. However, those who had missing data were more likely to be nurses in irregular shift work (χ^2^ = 11.85, df = 2, *p* < 0.01), and have higher work pressure (χ^2^ = 6.28, df = 2, *p* < 0.05).

The prevalence of depressive symptoms was compared between categories for each socio-demographical variable at T_1_ and shiftwork-related variable at T_2_ using chi-square test (see Table 1). Pearson correlation analysis was performed in main variables (including sleep reactivity, sleep disturbance, ERD, and depressive symptoms). Mediation analysis was conducted to test whether sleep disturbance at T_2_ and ERD at T_3_ mediated the relationship between sleep reactivity at T_1_ and depressive symptoms at T_4_. For this hypothesized model, current alcohol use, parents' marital quality, sleep disturbance at T_1_, ERD at T_1_, and depressive symptoms at T_1_ were included into the model as the covariates. Multiple fit indices including comparative fit index (CFI), Tucker-Lewis index (TLI), root mean square error of approximation (RMSEA), and standardized root mean square residual (SRMR) were used to assess the model fit (29). Based on bias-corrected bootstrapping with 5000 samples, we calculated the lower and upper values of the 95% confidence intervals (CIs) for all indirect effects. The effect was significant if the confidence intervals do not include zero. Missing data were handled with the full information maximum-likelihood estimation procedure (FIML). All data were analyzed using IBM SPSS version 26.0 and Mplus 7.4, with a significant α threshold of 0.05 (two-tailed).

**Table 1:** Characteristics of depressive symptoms at T_1_ and T_4_.

**Characteristics**	**Overall (T_**1**_)** ***n* (%)**	**Depressive symptoms at T**_**1**_				**Overall (T_**4**_)** ***n* (%)**	**Depressive symptoms at T**_**4**_			
		**No (%)**	**Yes (%)**	*****χ^2^*****	***p***		**No (%)**	**Yes (%)**	*****χ^2^*****	***p***
BMI	723			2.45	0.29	673			1.04	0.59
Underweight	270 (37.3)	224 (83.0)	46 (17.0)			249 (37.0)	197 (79.1)	52 (20.9)		
Normal	430 (59.5)	336 (78.1)	94 (21.9)			402 (59.7)	306 (76.1)	96 (23.9)		
Overweight	23 (3.2)	18 (78.3)	5 (21.7)			22 (3.3)	18 (81.8)	4 (18.2)		
Only child (siblings)	725			3.06	0.08	675			0.78	0.38
No	683 (94.2)	542 (79.4)	141 (20.6)			639 (94.7)	492 (77.0)	147 (23.0)		
Yes	42 (5.8)	38 (90.5)	4 (9.5)			36 (5.3)	30 (83.3)	6 (16.7)		
Residence	725			1.50	0.47	675			0.76	0.69
City	219 (30.2)	180 (82.2)	39 (17.8)			204 (30.2)	157 (77.0)	47 (23.0)		
Town	171 (23.6)	132 (77.2)	39 (22.8)			157 (23.3)	118 (75.2)	39 (24.8)		
Village	335 (46.2)	268 (80.0)	67 (20.0)			314 (46.5)	247 (78.7)	67 (21.3)		
Exercise habit	725			10.56	0.001	675			0.64	0.42
No	477 (65.8)	365 (76.5)	112 (23.5)			445 (65.9)	340 (76.4)	105 (23.6)		
Yes	248 (34.2)	215 (86.7)	33 (13.3)			230 (34.1)	182 (79.1)	48 (20.9)		
Current alcohol use	725			2.05	0.15	675			5.92	0.015
No	707 (97.5)	568 (80.3)	139 (19.7)			658 (97.5)	513 (78.0)	145 (22.0)		
Yes	18 (2.5)	12 (66.7)	6 (33.3)			17 (2.5)	9 (52.9)	8 (47.1)		
Parents' marital quality	724			22.89	<0.001	674			11.78	0.003
Poor	39 (5.4)	25 (64.1)	14 (35.9)			35 (5.2)	27 (77.1)	8 (22.9)		
Moderate	149 (20.6)	103 (69.1)	46 (30.9)			137 (20.3)	91 (66.4)	46 (33.6)		
Good	536 (74.0)	451 (84.1)	85 (15.9)			502 (74.5)	403 (80.3)	99 (19.7)		
Family income (/month)	724			5.07	0.08	674			2.41	0.30
< ¥5000	431 (59.5)	353 (81.9)	78 (18.1)			401 (59.5)	316 (78.8)	85 (21.2)		
¥ 5000 to ¥9999	226 (31.2)	180 (79.6)	46 (20.4)			213 (31.6)	163 (76.5)	50 (23.5)		
≥¥10000	67 (9.3)	47 (70.1)	20 (29.9)			60 (8.9)	42 (70.0)	18 (30.0)		
Shift schedule						642			1.01	0.60
Two shifts	NA	NA	NA			156 (24.3)	116 (74.4)	40 (25.6)		
Three shifts	NA	NA	NA			374 (58.3)	289 (77.3)	85 (22.7)		
Irregular	NA	NA	NA			112 (17.4)	89 (79.5)	23 (20.5)		
Night shift nap						642			3.18	0.07
No	NA	NA	NA			226 (35.2)	183 (81.0)	43 (19.0)		
Yes	NA	NA	NA			416 (64.8)	311 (74.8)	105 (25.2)		
Pressure during shiftwork						642			2.16	0.34
Mild	NA	NA	NA			211 (32.9)	155 (73.5)	56 (26.5)		
Moderate	NA	NA	NA			402(62.6)	316 (78.6)	86 (21.4)		
High	NA	NA	NA			29 (4.5)	23 (79.3)	6 (20.7)		

*BMI, body mass index; NA, Not applicable*.

## Results

### Participant Socio-Demographics

All recruited participants were females After they transferred into shiftwork, 24.3% of participants reported a rotating day-evening shift (two shifts) work schedule, 58.3% reported a rotating day-evening-night shift (three shifts) work schedule, and 17.4% reported an irregular shift work schedule. In addition, about 64.8% of participants had night shift naps, and 67.1% reported moderate or severe pressure during shift work. Compared with the prevalence of sleep disturbance before shift (poor sleep quality: 30.6%; DIS: 9.9%; DMS: 9.9%; EMA: 8.4%), it increased significantly after shift (poor sleep quality: 40.3%; DIS: 17.9%; DMS: 13.8%; EMA: 13.7%). Moreover, the paired-samples *t*-test showed that the mean level of sleep disturbance after shift was significantly higher than that before shift (*t* = 6.06, *df* = 689, *p* < 0.001). In terms of depressive symptoms, the prevalence of depressive symptoms (cut-off score ≥10) increased slightly (T_1_: 20.0% vs. T_4_: 22.7%). However, the paired-samples *t*-test showed that the mean level of depressive symptoms after shift was significantly higher than that before shift (*t* = 2.36, *df* = 674, *p* < 0.05). In addition, as shown in Table 1, current alcohol use and parents' marital quality were associated with depressive symptoms at T_4_, and thus these variables were adjusted as covariates in the subsequent analysis.

### Correlation for Main Measures

Descriptive statistics and correlations for main variables (including sleep reactivity, sleep disturbance, ERD, and depressive symptoms) were shown in Table 2. As expected, higher sleep reactivity (T_1_) was positively associated with higher sleep disturbance (T_2_), ERD (T_3_), and depressive symptoms (T_4_) (*p* < 0.001). Moreover, sleep disturbance (T_2_) and ERD (T_3_) were significantly and positively associated with depressive symptoms (T_4_) (*p* < 0.001).

**Table 2:** Descriptive statistics and correlations for main measures.

**Variables**	**M ± SD**	**1**	**2**	**3**	**4**	**5**	**6**	**7**
1. Sleep reactivity at T_1_	17.25 ± 5.58	1.00						
2. Sleep disturbance at T_1_	4.11 ± 2.98	0.26	1.00					
3. Sleep disturbance at T_2_	4.95 ± 3.23	0.20	0.32	1.00				
4. ERD at T_1_	32.85 ± 11.32	0.47	0.29	0.22	1.00			
5. ERD at T_3_	30.43 ± 12.02	0.27	0.21	0.27	0.40	1		
6. Depressive symptoms at T_1_^a^	5.82 ± 4.27	0.44	0.34	0.25	0.68	0.41	1	
7. Depressive symptoms at T_4_^a^	6.28 ± 5.22	0.23	0.16	0.29	0.36	0.43	0.44	1

a *Sleep-related item (item 3) was excluded from PHQ-9; ERD, Emotion regulation difficulties; All coefficients were statistically significant at p < 0.001*.

### Mediating Effects of Sleep Reactivity on Depression

First, the direct effect of sleep reactivity on depression was first examined in the model without mediators. The results indicated that depressive symptoms at T_4_ were significantly predicted by sleep reactivity at T_1_ (β = 0.21, *t*=5.45, *p* < 0.001). Second, after controlling for current alcohol use, parents' marital quality, sleep disturbance at T_1_, ERD at T_1_, and depressive symptoms at T_1_, the longitudinal mediation model was examined with sleep reactivity at T_1_ as the predicting variable, sleep disturbance at T_2_ and ERD at T_3_ as the serial mediators, and depressive symptoms at T_4_ as the outcome variable. To avoid overlap with sleep problems, sleep-related item (item 3: *Trouble falling or staying asleep, or sleeping too much*) of PHQ-9 was excluded in the mediation analyses. The hypothesized model demonstrated a good fit, χ^2^ = 41.26, *df* = 8, *p* < 0.001, CFI = 0.93, TLI = 0.88, RMSEA = 0.07, and SRMR=0.04.

**Figure 2:**
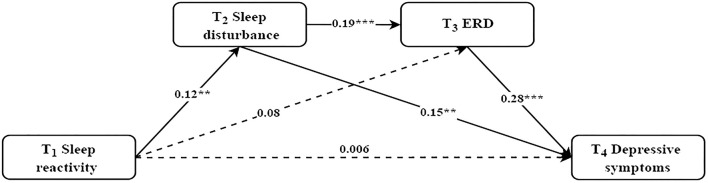
The mediating effect of sleep disturbance and ERD in the relationship between sleep reactivity and depressive symptoms. Controlled for current alcohol use, parents' marital quality, sleep disturbance at T_1_, ERD at T_1_, and depressive symptoms at T_1_. Standardized coefficients are reported; Dashed line indicates a non-significant coefficient; **p < 0.01, ***p < 0.001.

As shown in Figure 2, standardized regression coefficients displayed that the direct path from sleep reactivity at T_1_ to depressive symptoms at T_4_ was not significant (β = 0.006, *p* = 0.88). The path from sleep reactivity at T_1_ to sleep disturbance at T_2_ (β = 0.12, *p* < 0.01), and the path from sleep disturbance at T_2_ to ERD at T_3_ (β = 0.19, *p* < 0.001) and depressive symptoms at T_4_ (β = 0.15, *p* < 0.001), and the path from ERD at T_3_ to depressive symptoms at T_4_ (β = 0.28, *p* < 0.001) were all significant. The path from sleep reactivity at T_1_ to ERD at T_3_ was non-significant (β = 0.08, *p* = 0.06).

The bootstrap procedure (see Table 3) revealed a significant indirect effect with sleep disturbance as a mediator between sleep reactivity and depressive symptoms (β = 0.018, *SE* = 0.008, *p* < 0.05, 95% CI [0.005, 0.034]). Moreover, there was a significant serial mediation between sleep reactivity and depressive symptoms via increased sleep disturbance and sequentially increased ERD (β = 0.006, *SE* = 0.003, *p* < 0.05, 95% CI [0.002, 0.012]). The total indirect effect from sleep reactivity at T_1_ to depressive symptoms at T_4_ was statistically significant (β = 0.048, *SE* = 0.016, *p* < 0.01, 95% CI [0.018, 0.081]).

**Table 3:** Standardized direct and indirect path coefficients from sleep reactivity to depression.

	**β**	***SE***	***P-value***	**BC 95% CI**
**Direct effect from SR to Dep**				
T_1_ SR → T_4_ Dep	0.006	0.043	0.89	−0.076 to 0.088
Indirect effect from SR to Dep				
T_1_ SR → T_2_ SD → T_4_ Dep	0.018	0.008	0.017	0.005 to 0.034
T_1_ SR → T_3_ ERD → T_4_ Dep	0.023	0.013	0.071	0 to 0.051
T_1_ SR → T_2_ SD → T_3_ ERD → T_4_ Dep	0.006	0.003	0.019	0.002 to 0.012

*SR, Sleep reactivity; Dep, Depressive symptoms; SD, Sleep disturbance; ERD, Emotion regulation difficulties; β, standardized regression coefficient; SE, standard error; BC 95% CI, Bias-corrected 95% confidence interval*.

## Discussion

This study was the first longitudinal investigation in a cohort of student nurses, exploring the effect of sleep reactivity on depressive symptoms via the serial mediating roles of sleep disturbance and ERD. Our major findings include: (1) depressive symptoms were highly prevalent in student nurses after shift; (2) sleep disturbance played a longitudinal mediating role in the relation between sleep reactivity and depressive symptoms; and (3) sleep disturbance and ERD sequentially mediated the association between sleep reactivity and depressive symptoms. These findings advanced our understanding of depression in shift workers and might contribute to develop targeted interventions to reduce the risk of depression.

### The Prevalence of Depression

Nurses may encounter different stressors because of their special working environment. Thus, it is particularly important to pay attention to the depression of nurses. Depressed mood was found to be highly prevalent among nurses (30). More than one in five student nurses in the current study reported depressive symptoms after shift, which was similar with prior findings. For example, Xu et al. surveyed 763 college nursing students in China and found that 22.9% of participants reported high level of depression (31). In another study of 9789 female nurses from South Korea, researchers found that 26.8% of nurses reported moderate/severe depression (4). The high prevalence of depression found among nurses may be explained by two reasons. On the one hand, student nurses who are in the transition from late adolescence to young adulthood may be more susceptible to depression (32, 33). On the other hand, females may be more likely to ruminate than males when facing tremendous pressures and challenges (34), which may increase the risk of depression.

### Mediating Role of Sleep Disturbance Between Sleep Reactivity and Depressive Symptoms

The present study found that sleep disturbance played a longitudinal mediating role in the relation between sleep reactivity and depressive symptoms. In particular, student nurses with greater sleep reactivity tend to exhibit acute sleep-disruptive responses to circadian rhythm misalignment, and suffer from more sleep disturbances after taking up shiftwork, which in turn was associated with greater depressive symptoms. Previous cross-sectional studies have revealed that elevated sleep reactivity and insomnia additively or synergistically aggravate depressive symptoms (35), and insomnia partially mediated the association between sleep reactivity and depressive symptoms (8). Moreover, in a longitudinal study with 96 normal sleeping non-shift workers, researchers found that high sleep reactivity before shift work increased risk for shift work disorder, which led to more severe depressive symptoms after transiting into rotating shifts (7). According to the 3P model of insomnia (36), this study provided further supporting that sleep reactivity was a reliable predisposing factor of sleep disturbance. The mechanisms between this relationship are still unclear but may be understood via the following aspects. First, individuals with high sleep reactivity might exhibit a high level of stress-induced cognitive intrusion (6), which have been evidenced to be associated with insomnia. Second, sleep reactivity has been shown to be significantly linked to dysfunctional beliefs and attitudes about sleep (37), leading to sleep disturbance (38, 39). Third, neuroscience research suggest that high sleep reactivity may reflect a neurological deficit in which the arousal mechanisms cannot be effectively weakened, thus manifesting in the tendency toward wakefulness before and during the sleep periods (7). Further researches are warranted to examine the prospective associations and cognitive neuropsychological mechanisms between sleep reactivity and sleep disturbance. In addition, the present study also showed that greater sleep disturbances may subsequently lead to increased depression, which has been confirmed in a previous meta-analysis (40).

### Mediating Role of Sleep Disturbance and ERD Between Sleep Reactivity and Depressive Symptoms

Student nurses may not only have to adapt to the shift work schedules but also need to fit in the whole new working environment (e.g., communicating with their leaders, patients, etc.), all of which may make them nervous and afraid. Therefore, good emotional regulation skills are necessary for them to cope with these stressors. Another noteworthy finding of our study was that sleep disturbance and ERD sequentially mediated the association between sleep reactivity and depressive symptoms. To be specific, student nurses with high sleep reactivity may experience more severe sleep disturbances, which was linked to subsequent increased difficulties in obtaining effective emotion regulation strategies, and further led to increased depression. Growing evidence have suggested that sleep disturbance can impair individuals' ability to regulate negative emotions (16, 41) and enhance impulsivity to negative stimuli (42), which may lead to mood instability and depression. Our findings were consistent with previous studies in clinical patients (43) and normal population (18, 19). For example, in a sample of 880 firefighters from United States, researchers found that difficulties in emotion regulation, specifically limited access to emotion regulation strategies, played a mediating role between sleep disturbance and depression symptoms (18). In addition, student nurses in this study were now at the end of adolescence when they would experience the integration of top-down control of emotional processes and susceptibility to sleep difficulties (44), which together increased the risk of mental health problems. Therefore, sleep disturbance and ERD, two modifiable risk factors, should be given more attention in the intervention and prevention of depression, especially for student nurses with high sleep reactivity.

Sleep reactivity at T_1_ did not independently predict depressive symptoms at T_4_ after considering the effects of sleep disturbance and ERD. The current results were in line with Palagini et al. (9), but were inconsistent with Nakajima et al. (35) and Vargas et al. (8). Given that most of these studies are cross-sectional, it is impossible to explore whether sleep reactivity predict changes in depressive symptoms. Moreover, additional large-scale prospective studies are needed because of the currently mixed results.

### Strengths and Limitations

The present study had several strengths, including a four-wave longitudinal design with large sample sizes, and the strict control of demographic covariates and all studied variables at baseline. However, some limitations should also be considered. First, all measures were based on self-report methods instead of objective assessments or clinical diagnostic evaluations, which may be prone to reporting bias and may inflate the associations among variables due to shared method variance. Future studies are suggested to employ multiple methods to reduce the influence of recall bias. Second, the generalizability of the findings is limited by the highly homogeneous sample (female, young age, and student nurses). It is still uncertain whether these findings could be generalized to male healthcare shift workers, shift workers in other occupational settings, or shift workers at different ages. Future studies should put efforts into confirming this important issue. Third, substance use, such as tobacco and caffeine, has positively correlation with sleep disturbance and depressive symptoms. Although this is not a main concern of the present study, it deserves future research to investigate. Last but not least, shift workers also commonly report anxiety and fatigue symptoms, thus, we suggest that further work is needed to draw attention to these psychological problems.

### Clinical Implication

From a public health perspective, our findings have important implications for nurses and nursing managers. First, the early detection of high sleep reactivity holds potential to prevent the development of mood pathology (6). As a prognostic marker of future psychiatric illness, sleep reactivity may identify nurses who are at-risk for subsequent depression. Therefore, nurses with high sleep reactivity may be a targetable population for preventive efforts against depression. Hospital administrators can use scientific evaluation tools in the recruitment process, potentially screening out shift workers with low sleep reactivity, to improve the staff-post matching accuracy. Second, intervention and treatments of sleep disturbance may be helpful in reducing the risk of depression to nurses with high sleep reactivity. Previous studies have found that cognitive behavioral therapy for insomnia (CBT-I) can help individuals improve their sleep quality and relieve depressive symptoms (45, 46). Third, the development of effective emotion regulation programs among nurses may contribute to preventing depression. Thus, it is very important to coach them on effective emotion regulation strategies, such as cognitive reappraisal and acceptance of emotions, to regulate and express their negative emotions effectively.

## Conclusion

In conclusion, this prospective study provides evidence of a temporal relationship between sleep reactivity and depressive symptoms in student nurses, with sleep disturbance and ERD as serial mediators. Sleep reactivity might be considered as an indicator of shiftwork adaptability in the recruitment evaluation. Furthermore, psychological health recommendations for depression prevention focusing on improving nurses' sleep problems and their abilities to regulate emotions are needed.

## Data Availability Statement

The de-identified dataset used and/or analyzed during the current study are available from the corresponding author on reasonable request.

## Ethics Statement

This study was approved by the Human Research Ethics Committee of South China Normal University, Guangzhou, China. Written informed consent to participate in this study was provided by the participants' legal guardian/next of kin.

## Author Contributions

XS study design, data analysis and paper revision. HQ data analysis and paper revision. SW, ZiL, and ZhL paper revision. FF study design. All authors contributed to the article and approved the submitted version.

## Funding

The study was funded by the National Natural Science Foundation of China (Grant No. 31900789, 31871129), and the Advanced Talents Incubation Program of the Hebei University (Grant No. 521000981309).

## Conflict of Interest

The authors declare that the research was conducted in the absence of any commercial or financial relationships that could be construed as a potential conflict of interest.

## Publisher's Note

All claims expressed in this article are solely those of the authors and do not necessarily represent those of their affiliated organizations, or those of the publisher, the editors and the reviewers. Any product that may be evaluated in this article, or claim that may be made by its manufacturer, is not guaranteed or endorsed by the publisher.
